# Dietary and competition effects on life history attributes of *Chrysomya megacephala* and *Lucilia sericata* (Diptera: Calliphoridae) in south-west Europe

**DOI:** 10.1007/s00414-025-03425-1

**Published:** 2025-01-23

**Authors:** A. Martínez-Sánchez, T. Ivorra, Y. Velásquez, L. Cerdá-Ortega, C. Ibáñez, S. Rojo

**Affiliations:** 1 University of Alicante, Department of Environmental Sciences and Natural Resources, PO Box 99, Alicante, E-03080 Spain; 2 Universiti Malaya, Department of Parasitology, Faculty of Medicine, Kuala Lumpur, 50603 Malaysia; 3 Department Environmental Sciences and Natural Resources, Faculty of Sciences III, University of Alicante, Carretera San Vicente del Raspeig s/n, San Vicente del Raspeig, Alicante, 03690 Spain

**Keywords:** Annual activity, Forensic entomology, Larval competition, Larval substrates, Life cycle, Spain

## Abstract

The range of the oriental latrine fly (*Chrysomya megacephala*) is currently expanding. It coexists with another blowfly with a similar ecology, the green bottle fly (*Lucilia sericata*), one of the most abundant species in carrion during warm months. It is essential to understand the influence of temperature, larval substrate type, and larval competition on the development rates of these necrophagous calliphorids to evaluate the role and the adaptation of *C. megacephala* in the ecosystems, and on the necrophagous Diptera community. Moreover, due to the forensic importance of both species, biological data are need for forensic purposes in south-west Europe. This study represents the first on the biology of *C. megacephala* in Europe, and of *L. sericata* in Spain. We performed a field experiment in Alicante province (south-east Spain) to analyse the monthly abundance of both species in natural conditions, and three lab rearing experiments involving both species to evaluate (i) development at constant temperatures, (ii) the growth of larvae and pupae on different substrates, and (iii) coexistence in pure and mixed cultures under controlled abiotic conditions. The results allowed us to analyse the biological differences between both species, and their effects on the forensic entomology application.

**Supplementary Information:**

The online version contains supplementary material available at 10.1007/s00414-025-03425-1.

## Introduction

Necrophagous species of Calliphoridae (Diptera) play a key role in cadaveric decomposition [1]. In south-west Europe, the principal blowflies on corpses belong to the genera *Calliphora* Robineau-Desvoidy, 1830, *Lucilia* Robineau-Desvoidy, 1830, and *Chrysomya* Robineau-Desvoidy, 1830 [2]. The presence of the oriental latrine fly – *Chrysomya megacephala* (Fabricius, 1794) (Diptera: Calliphoridae) – is recent in Europe, with its distribution still restricted to Spain, Portugal, and Malta [3–7]. The developmental stages of these dipterans are directly affected by temperature, larval substrates, larval competition, and geographic origin [8–11]. In fact, in Mediterranean countries, there are variations in biological traits between populations of the same species with a wide distribution [10, 12, 13]. On the other hand, larval competition is the most important occurrence on a corpse [14–18], and the consequences can be seen in the larval size, the mortality rate, and the longevity of stages during the life cycle.

To evaluate any entomological evidence, it is essential to know the sarcosaprophagous fauna composition of the study area [19]. *Chrysomya megacephala* is native to the Oriental and Australasian regions, and its distribution has expanded to all continents since the 1970s: it is considered an invasive species [20–23]. It is synanthropic and necrophagous on different dead tissues, so its potential use as forensic evidence is obvious [24–27]. Indeed, it has been reported on human corpses from Asia [24, 25, 28, 29], South America [30, 31], and Africa [32, 33]. On the other hand, the green bottle fly *Lucilia sericata* (Meigen, 1826) (Diptera: Calliphoridae) is a native species with a cosmopolitan synanthropic distribution. It is regarded as one of the first coloniser species during spring and summer in south-west Europe [34–37]. Differences in their life cycle span have been observed depending on the temperature, type of substrate, and origin of the population, and these factors must be considered when the minimum post-mortem interval (mPMI) is being estimated [10, 38, 39].

Forensic entomology requires biological data that, ideally, is obtained from local populations [19], which are the basic parameters to estimate the mPMI, using isomorphen diagrams, larval growth curves, and accumulated degree days (ADDs). The extrapolation of data from different populations should be considered with caution. However, there is no biological information about *C. megacephala* in Europe, while *L. sericata* displays population differences, but in southern Europe, not much is known about its biology [10, 40–42].

Given the aforementioned background, the main objective of this study was to gain crucial information about the development of *C. megacephala* and *L. sericata* from local populations to understand more about the ecology of these two species with forensic importance. The specific aims were: (i) to compare the adult annual activity of both species in south-east Spain and to confirm their coexistence; (ii) to obtain the duration of developmental stages of these species from south-west Europe at different constant temperatures; (iii) to evaluate their life cycles on the basis of larval and intrapuparial development with two different larval diets; and (iv) to study their mortality rates and immature stage periods in intra- and interspecific competition trials under controlled conditions. The results of this ecological study will increase knowledge about both blowflies in a specific geographical area and provide preliminary data about *C. megacephala* in Europe, using a population originating from this continent.

## Materials and methods

### Annual activity

To understand the annual activity of *C. megacephala* and *L. sericata* and whether they co-occur, a field sampling was carried out in a wooded area on the campus of the University of Alicante (San Vicente del Raspeig, south-east Spain; 38°37’98"N, 0°52’69"W), from September 2020 to December 2021. The campus, between the towns of Alicante and San Vicente, is an urban landscaped environment, with low human presence, and bordered by several access roads. The place where the traps were placed is a hemisynanthropic habitat, with artificial forest mass of *Pinus halepensis* (Mill., 1768), some isolated specimens of *Pistacia lentiscus* (L., 1753), small patches of *Lygeum spartum* (L., 1754), and some wildflowers. Around 50 m away from this area is an artificial lake with ducks, which serves as a tourist attraction for university community. Every month, three adult traps (wind-oriented traps [WOTs]) baited with 300 g of fresh fish, at a height of 1.3 m above the ground and at a straight line 20 m from each other, were active for 72 h. The traps were located under the shade of pines, in a fenced plot, and, therefore, with restricted access. The area of study is included within the semi-arid thermo-Mediterranean bioclimate region. During the sampling period, temperature and humidity were recorded daily from the weather station closest to the experimental site, located on the campus of the University of Alicante, 500 m from the traps (weather station Sant Vicente – IIG UA). The collected specimens were frozen and separated by morphotype, and the species were identified using keys provided by Rognes [43, 44] and Peris and Gonzalez-Mora [45]. All specimens were preserved in vials with 70% ethanol and deposited at the Entomological Collection of the University of Alicante, Department of Environmental Sciences and Natural Resource (CEUA).

### Temperature effect experiment

Larval development was evaluated at 18, 23, and 28 °C for both species in environmental chamber with a temperature control (0.1 °C precision; Equitec, Mod. Eichs HR, Research Technical Services, University of Alicante). For this purpose, permanent colonies of *C. megacephala* and *L. sericata* were established in 2017 from local specimens captured in the campus of the University of Alicante – by using odour-baited traps (WOTs) for *L. sericata* adults and on a pig carcass for *C. megacephala* larvae – and kept in the laboratory under controlled conditions (25 ± 2.49 °C, 50–60% relative humidity, and a 12-h photoperiod), without reintroduction of wild specimens. The adults were kept in mesh cages (40 × 40 × 40 cm; density around 300 individuals) with water and sugar available *ad libitum*, and pork liver as the oviposition and larval medium.

The eggs were obtained over a period of 3 h. Approximately 100 neonate larvae were placed on pork liver cut into approximately 1-cm^3^ cubes (200 g at one time), available *ad libitum*. These pieces were put into a plastic container and then placed in a larger container with sawdust at the bottom for pupation, covered with mesh. These containers were placed in an environmental chamber at each of the constant temperatures proposed, with 60–70% relative humidity and 14-/10-h photoperiod; there were 10 replicates for each temperature and species. Every 24 h, 5 larvae were collected randomly from each replicate, weighed alive with an analytical balance (Acculab-VI-1200, 0.1 mg precision), killed in boiling water, and stored in 70% ethanol. The preserved larvae were measured using a digital calliper (0.01 mm precision) and the larval instar (LI, LII, and LIII) was identified based on the slits in the posterior spiracles [26] by using binocular microscopy (Leica M80). For the third instar larvae, it was noted whether they were at the feeding or post-feeding stage (when larvae have abandoned food to pupate). The duration of each stage or instar was determined when at the least 10% of the individuals changed to the next phase; this value was averaged over the 10 replicates. The isomorphen diagrams for *C. megacephala* and *L. sericata* were produced by plotting the egg, larval, and pupal development times at constant temperatures, and the ADDs were calculated with the equation ADD = y (t – t_0_), where *y* is the development time (days), *t* is the rearing temperature, and *t*_*0*_ is the lower development threshold temperature (°C), also referred to as D_0_ and the lower developmental threshold (LDT), which is considered the species minimum temperature of development. Previously, *t*_*0*_ for *C. megacephala* and *L. sericata* was estimated based on linear regressions of the developmental rates (y = 1/development time) at constant temperatures.

### Larval diet experiment

The same methodology used to measure the temperature effects on larval development was used to evaluate the dietary effects on larval and intrapuparial development under controlled laboratory conditions (25 ± 2.49 °C, 50–60% relative humidity, and a 12-h photoperiod). Two different sources were tested as larval substrates (200 g of each, available *ad libitum*), with three replicates per species: (i) pork liver, because it is common substrate to rear larvae in forensic research and easy to compare the data from other geographical regions, and (ii) minced pork, because it is a combination of different necrophagous tissues. Each stage and larval instar period were recorded described for the temperature effect experiment, and the length of development for the three larval instars for each replicate was measured each day. Then, from the first day of pupation, 10 pupae of each replicate were collected randomly and kept individually in Petri dishes. The live pupal weight was determined daily using an analytical balance (Acculab-VI-1200, 0.1 mg precision) until adult emergence.

### Larval competition experiment

Finally, to analyse possible larval competition between *C. megacephala* and *L. sericata* under laboratory conditions (23 ± 1 °C, 60–70% relative humidity, and a 12-h photoperiod), groups of neonatal larvae (1 day old) were tested in intraspecific (pure) and interspecific (mixed, 1:1 species ratio) conditions. Four larval densities were used (50, 100, 200, and 500 specimens), together with five replicates for each density and treatment. Each group of larvae was placed on 15 g of pork liver in a plastic pot covered with a fine mesh for 3 days to prevent the larvae from escaping. After the feeding period, the larvae were allowed to pupate in sawdust placed in the bottom of another larger pot. Then, the pupae were collected, and once the adults had emerged and expanded their wings, they were frozen, sexed, and counted. The total mortality rate was calculated based on the number of adults that emerged with respect to the initial number of larvae. The total immature period was calculated using the data obtained for each replicate from the neonatal larval time to adult emergence (at least 10% of individuals based on the density used). To ensure accuracy, each vial was checked every 24 h until the end of the experiment.

### Statistical analysis

The normality and homogeneity of variance of the data were tested using the Kolmogorov–Smirnov test. When the data were normally distributed, analysis of variance (ANOVA) was performed. When the data did not meet the normality assumption, a Kruskal–Wallis H test was used. If a significant difference was found, then Dunn’s or Tukey’s *post hoc* test was performed for pairwise comparisons. In the case of the competition experiments, the parametric data were analysed using ANOVA and a t-test, followed by the Holm–Sidak *post hoc* test. A p-value < 0.001 (or < 0.05, only in the case of pupal weight) was considered to indicate a statistically significant difference.

## Results

### Annual activity

**Fig. 1:**
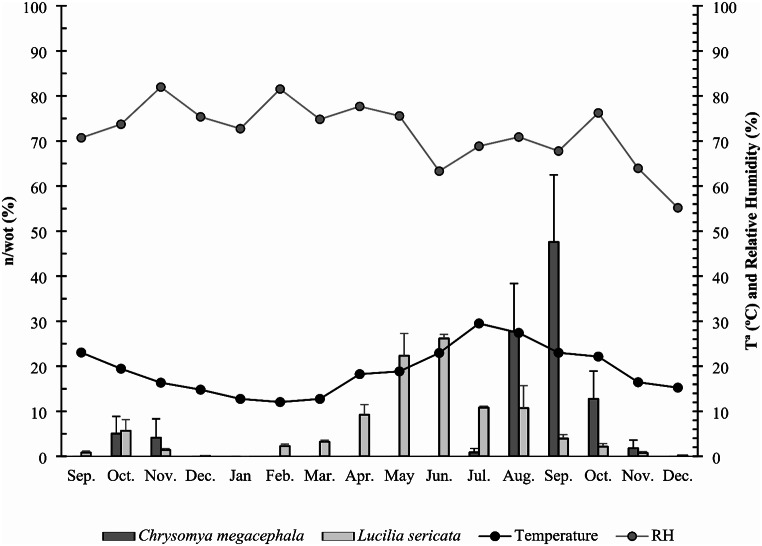
The monthly distribution of relative abundance of individuals per trap of *Chrysomya megacephala* and *Lucilia sericata* during the sampling period (2020–2021) in south-east Spain. The lines show the average temperature (°C) and relative humidity (%) in each month. WOT: wind-oriented trap

We captured four blowfly species: *Chrysomya albiceps* (Wiedemann, 1819) (*n* = 2487), *Calliphora vicina* Robineau-Desvoidy, 1830 (*n* = 1394), *L. sericata* (*n* = 1146), and *C. megacephala* (*n* = 83). The adult activity of *L. sericata* and *C. megacephala* during the sampled period is shown in Fig. 1. *Lucilia sericata* was present in all the months except January, peaking in spring (June, 26.17% of individuals); *C. megacephala* was collected from July to November, with a peak in summer (September, 46.67% of individuals). Both species coexisted during the summer months, avoiding the hottest month (July, average 26.25 °C, maximum 37.10 °C), but when the presence of *L. sericata* began to decrease, the presence of *C. megacephala* increased gradually. During this period, *L sericata* and *C. megacephala* peaked at 23 °C and were practically absent below 16 °C. Temperature, humidity, and competition with the other blowfly species collected in summer, such as the predator *C. albiceps*, could be related to these trends. There was an increase in *L. sericata* activity when the temperature increased during the spring, and an increase in *C. megacephala* activity during autumn, when the temperature decreased. *Lucilia sericata* peaked at the minimal humidity registered during the sampling period, that is, after spring rain (June, 23.28 °C and 63.31% relative humidity); *C. megacephala* peaked when the relative humidity increased due to the first autumn rain (September, 23.67ºC and 67.78% relative humidity). In the case of *C. albiceps*, activity was concentrated from June to September, coexisting with both *C. megacephala* and *L. sericata*.

### Temperature effect experiment

We calculated the average minimum egg, larval, and pupal development times for each species at constant temperatures (Table 1). The complete development was from 26.7 ± 0.5 days at 18 °C to 9.00 ± 0.0 days at 28 °C for *C. megacephala*, and from 31.1 ± 1.0 days at 18 °C to 13.6 ± 0.5 days at 28 °C for *L. sericata*. It decreased significantly in both species when temperature increased (*C. megacephala*: H_2,150_ = 140.40, *p* < 0.001; *L. sericata*: H_2,150_ = 135.17, *p* < 0.001). In addition, the larval (*C. megacephala*: H_2,150_ = 143.82, *p* < 0.001; *L. sericata*: H_2,150_ = 131.96, *p* < 0.001) and intrapuparial periods (*C. megacephala*: H_2,150_ = 139.25, *p* < 0. 001; *L. sericata*: H_2,150_ = 136.26, *p* < 0.001) decreased significantly when temperature increased. Regarding each larval instar, in general we observed the same relation between development and temperature in both species: when the temperature increased, the development time decreased (Table 1).

**Table 1:** Minimum period (mean ± standard error) of developmental stages and accumulated degree-days for *Chrysomya megacephala* and *Lucilia sericata* at different constant temperatures

	Average minimum period (days)					
Stage	*Chrysomya megacephala*			*Lucilia sericata*		
	18ºC	23ºC	28ºC	18ºC	23ºC	28ºC
Egg	2.0 ± 0.0	1.0 ± 0.0	1.0 ± 0.0	2.0 ± 0.0	1.0 ± 0.0	1.0 ± 0.0
Larva	14.8 ± 0.8^a^	6.0 ± 0.0^b^	4.0 ± 0.0^c^	16.9 ± 1.0^a^	6.8 ± 0.4^b^	6.0 ± 0.0^c^
L I instar	2.0 ± 0.0	1.6 ± 0.5	1.0 ± 0.0	1.0 ± 0.0	1.0 ± 0.0	1.0 ± 0.0
L II instar	2.5 ± 0.5	1.4 ± 0.5	1.0 ± 0.0	3.1 ± 0.5	2.0 ± 0.3	1.0 ± 0.0
L III instar	10.3 ± 0.8	3.0 ± 0.0	2.0 ± 0.0	12.7 ± 0.1	3.8 ± 0.3	4.0 ± 0.0
LIII feeding	6.8 ± 0.4	2.0 ± 0.0	1.0 ± 0.0	5.2 ± 0.5	2.0 ± 0.0	3.0 ± 0.0
LIII post-feeding	3.5 ± 0.5	1.0 ± 0.0	1.0 ± 0.0	7.5 ± 0.9	1.8 ± 0.3	1.0 ± 0.0
Pupa	9.9 ± 0.7^a^	6.2 ± 0.9^b^	4.0 ± 0.0^c^	12.2 ± 0.6^a^	9.0 ± 0.6^b^	6.6 ± 0.5^c^
Egg to adult	26.7 ± 0.5^a^	13.2 ± 0.9^b^	9.0 ± 0.0^c^	31.1 ± 1.0^a^	16.8 ± 0.8^b^	13.6 ± 0.5^c^
ADD total	139.37	134.90	136.98	254.82	222.10	247.79

^abc^ Different letters indicate significant differences between temperatures for each species (*p* < 0.001)

**Fig. 2:**
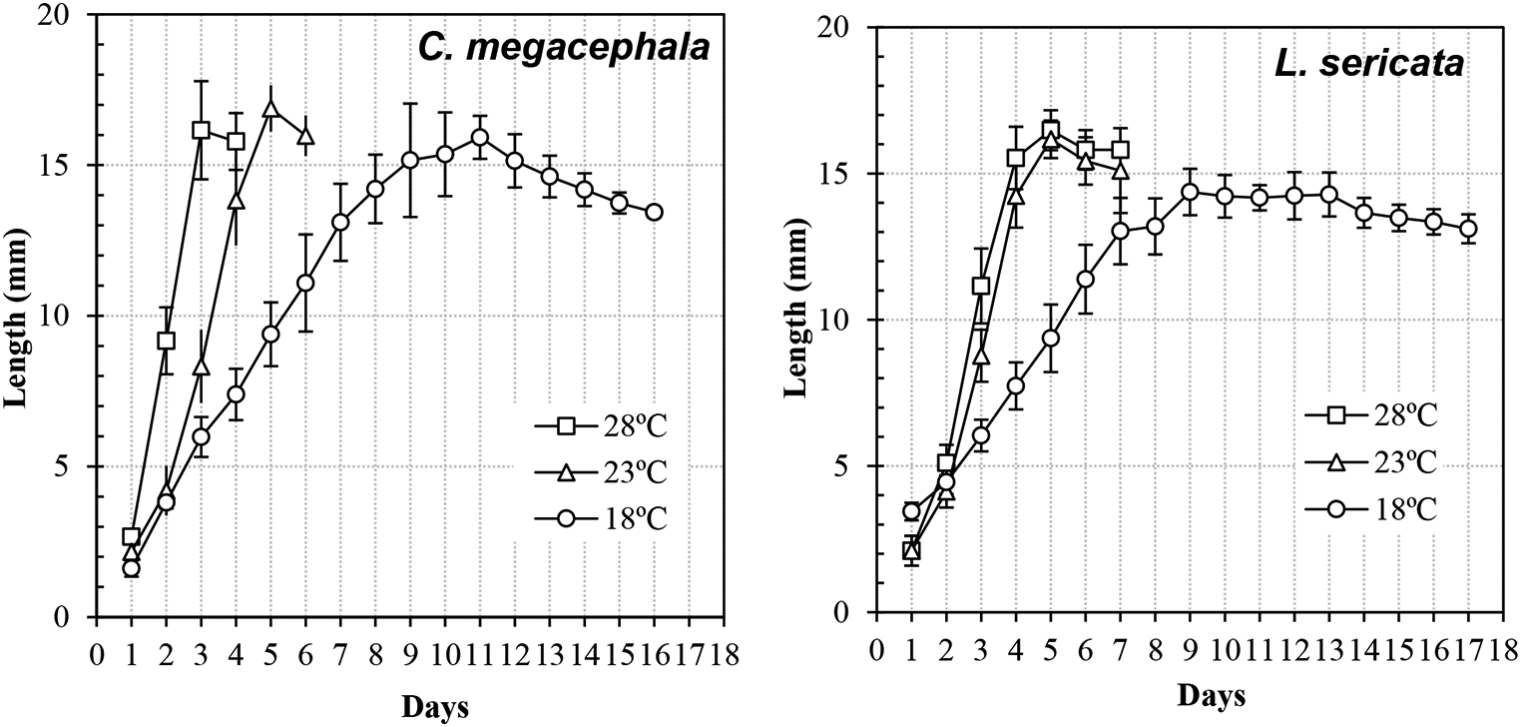
Growth curves (mean ± standard error of the mean length) of *Chrysomya megacephala* and *Lucilia sericata* larvae at different constant temperatures

**Fig. 3:**
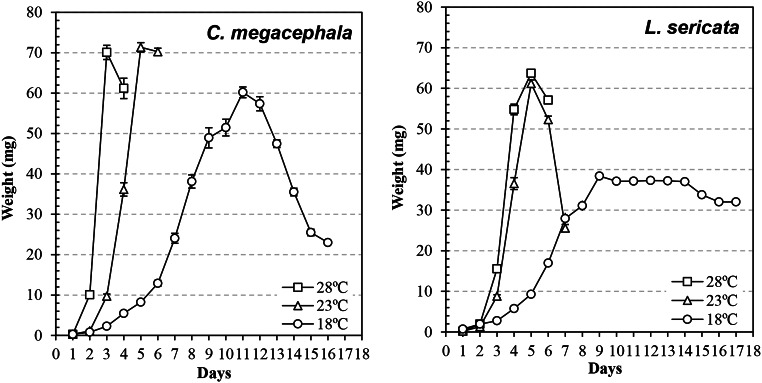
Growth curves (mean ± standard error of the mean weight) of *Chrysomya megacephala* and *Lucilia sericata* larvae at different constant temperatures

The larval length and weight at the three constant temperatures are shown in Figs. 2 and 3, respectively. The correlation between the weight and length was positive and significant in both species (*C. megacephala*: r^2^ = 0.97, *p* < 0.001; *L. sericata*: r^2^ = 0.97, *p* < 0.001). Temperature had a significant effect on larval development, and when temperature increased the larval length (*C. megacephala*: F_2,1103_ = 295.01, *p* = 0.001; *L. sericata*: F_2,1279_ = 499.14, *p* = 0.001) and larval weight (*C. megacephala*: F_2,1103_ = 320.09, *p* = 0.001; *L. sericata*: F_2,1279_ = 369.03, *p* = 0.001) increased.

The maximum length of *C. megacephala* was similar at all temperatures: 16.72 ± 1.80 mm at 28 °C, 16.88 ± 0.78 mm at 23 °C, and 15.92 ± 0.71 mm at 18 °C (*p* > 0.001; Fig. 2). For *L. sericata*, the maximum length was similar at 23 and 28 °C (*p* > 0.001) – 16.17 ± 0.64 mm and 16.48 ± 0.68 mm, respectively – but at 18 °C the larvae were shorter (*p* < 0.001), measuring 13.94 ± 0.92 mm (Fig. 2). When we analysed the length for both species, we observed that the slopes of the curves were almost vertical and equivalent at the initial moments at the two higher temperatures (23 and 28 °C), and this slope was relatively flat at the lowest temperature (18 °C), so the rate of growth decreased. The slopes for the exponential phase of growth (the period before the maximum length was reached) peaked on day 5 for *L. sericata* at 28 and 23 °C; in *C. megacephala*, the peak was day 3 for 28 °C and day 5 for 23 °C. Thus, at the highest temperatures *C. megacephala* lacked a stationary phase (the period after peak until pupation), indicating fast growth, but *L. sericata* had a short stationary phase, indicating a post-feeding period in which larvae migrated to begin metamorphosis. However, at 18 °C, the peak was around day 10 in both species, with a long stationary phase until the end of the larval stage.

The species were heavier at 23 and 28 °C (*C. megacephala*: 71.35 ± 8.17 mg and 69.12 ± 14.79 mg, respectively; *L. sericata*, 61.19 ± 6.19 mg and 63.70 ± 5.92 mg, respectively) compared with 18 °C (*C. megacephala*: 60.20 ± 9.39 mg; *L. sericata*: 35.61 ± 7.59 mg). The temperature effect was particularly pronounced for *L. sericata*. The variation in weight before pupation also differed based on temperature: for *C. megacephala*, the weight was 24.72 ± 3.97 mg at 18 °C, 70.25 ± 8.33 mg at 23 °C, and 61.18 ± 17.84 at 28 °C; for *L. sericata*, the weight was 30.49 ± 3.98 mg at 18 °C, 25.58 ± 6.27 mg at 23 °C, and 57.11 ± 5.20 at 28 °C. Before pupation, the weight dropped at 18 °C for *C. megacephala*; for *L. sericata*, the weight dropped at 18 and 23 °C. For the other cases, the weight changed little from the peak to pupation.

Finally, with limitations because only three temperatures were tested in this experiment, we used the development rates to estimate ADDs (Table 1) and to build the isomorphen diagrams (Supplementary Information, Fig. I). First, we had to calculate *t*_*0*_, plotting the development rates (*C. megacephala*: 0.04, 0.08, and 0.11 at 18, 23, and 28 °C, respectively; *L. sericata*: 0.03, 0.06, and 0.07 at 18, 23, and 28 °C, respectively). We obtained a regression equation (*p* < 0.001) for *C. megacephala* (y = 0.0074.*t*_*0*_ – 0.0946; r² = 0.999) and *L. sericata* (y = 0.0041.*t*_*0*_ − 0.0401; r² = 0.966). Thus, *t*_*0*_ was 12.78 °C for *C. megacephala* and 9.78 °C for *L. sericata.*

### Larval diet experiment

**Fig. 4:**
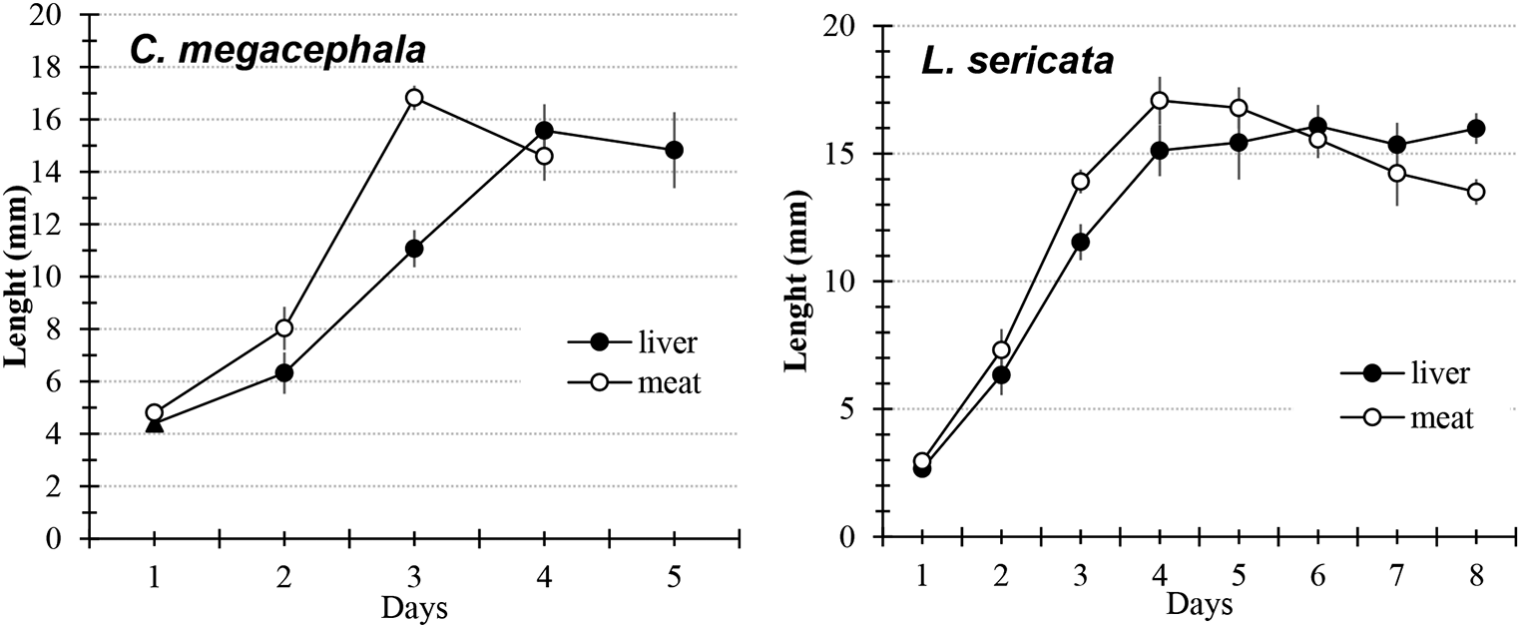
Larval length (mean ± standard deviation) of *Chrysomya megacephala* and *Lucilia sericata* fed on pork liver and minced pork meat under lab conditions (25 ± 2.49 °C, 50–60% relative humidity, and a 12-h photoperiod)

The development time was not affected significantly by the larval diet. In general, *C. megacephala* spent 10–12 days and *L. sericata* spent 16–17 days (Table 2). Regarding the larval size, for *C. megacephala* length was similar for both diets (*p* > 0.05), but diet affected the size of *L. sericata*. Specifically, larvae fed on minced pork meat were larger (*p* = 0.05) and reached their maximum length earlier than those reared on liver (Fig. 4): day 4 for minced pork meat (17.07 ± 0.93 mm) and day 6 for liver (16.08 ± 1.10 mm). At the final the larval stage, the larval length was equal for both diets (*p* = 0.609). For *C. megacephala*, maximum length was reached on day 3 when fed on minced pork meat (16.88 ± 1.38 mm) and day 4 when fed on liver (15.57 ± 0.93 mm); pupation began 1 day earlier for minced pork meat compared with liver as a diet. However, before pupation, the *C. megacephala* larval length was the same for both diets (*p* > 0.05; Fig. 4).

**Table 2:** Minimum period (mean ± standard error) of developmental stages for *Chrysomya megacephala* and *Lucilia Sericata* fed on pork liver and minced pork meat under lab conditions (25 ± 2.49 °C, 50–60% relative humidity, and a 12-h photoperiod)

	Minimum period (days)			
Stage	*Chrysomya megacephala*		*Lucilia sericata*	
	liver	meat	liver	meat
Egg	1.0 ± 0.0	1.0 ± 0.0	1.0 ± 0.0	1.0 ± 0.0
Larva	5.0 ± 0.0^a^	4.0 ± 0.0^a^	6.7 ± 0.3^b^	6.0 ± 0.0^b^
LI instar	0.3 ± 0.3	0.3 ± 0.3	1.0 ± 0.0	0.7 ± 0.3
LII instar	1.7 ± 0.3	1.3 ± 0.3	1.0 ± 0.0	1.3 ± 0.3
LIII instar	3.0 ± 0.0	2.3 ± 0.3	4.7 ± 0.3	4.0 ± 0.0
LIII feeding	2.0 ± 0.0	1.3 ± 0.3	2.7 ± 0.6	3.0 ± 0.0
LIII post-feeding	1.0 ± 0.0	1.0 ± 0.0	2.0 ± 0.0	1.0 ± 0.0
Pupa	5.7 ± 0.3^a^	5.0 ± 0.0^a^	9.0 ± 0.0^b^	8.7 ± 0.3^b^
Egg to adult	11.7 ± 0.3^a^	10.0 ± 0.0^a^	17.0 ± 0.0^b^	15.7 ± 0.3^b^

^abc^ Different letters indicate significant differences (*p* < 0.001)

**Fig. 5:**
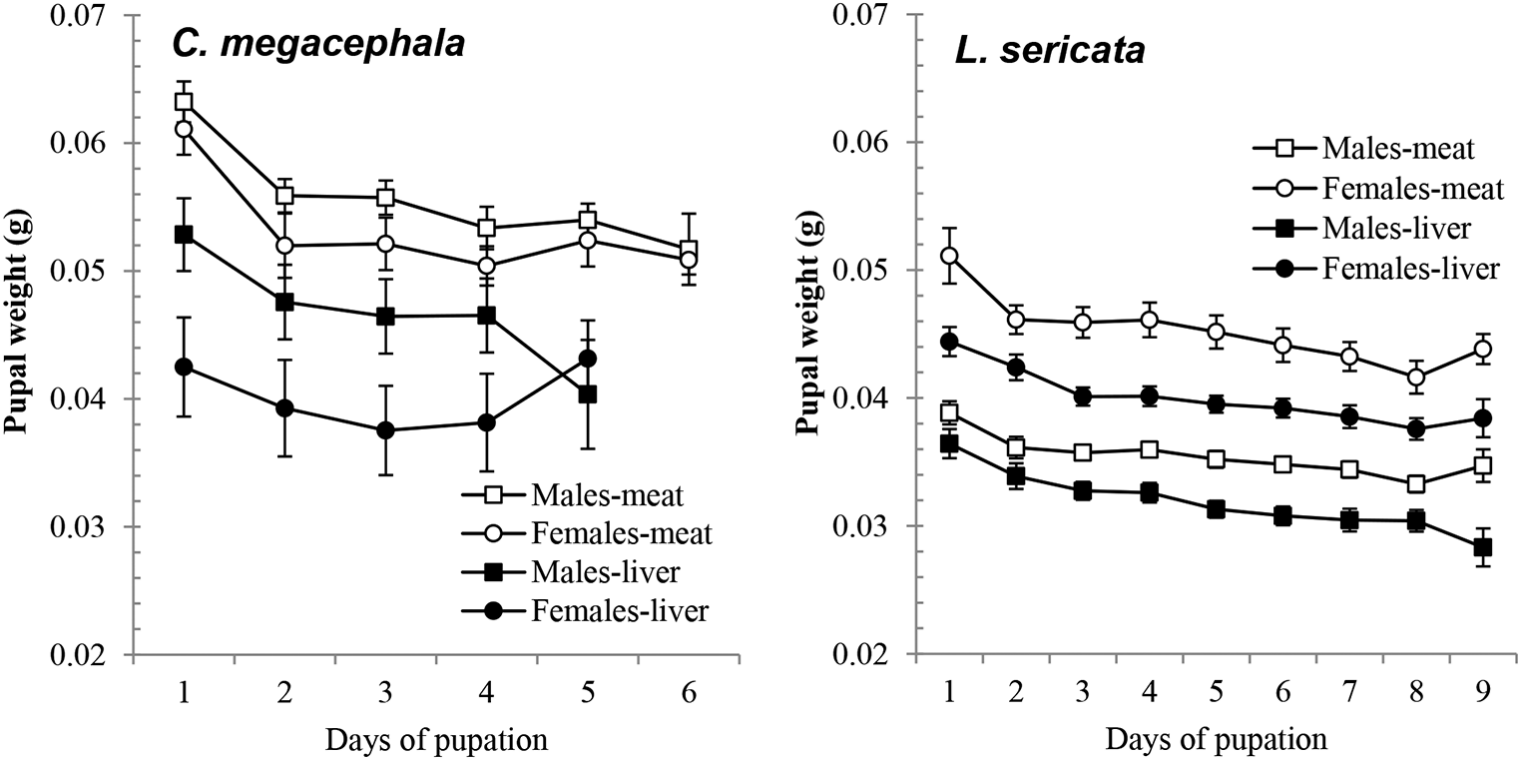
Average pupal weight of males (black) and females (white) (mean ± standard error of the mean) of *Chrysomya megacephala* and *Lucilia sericata* reared on pork liver (squares) and minced pork meat (circles) larval diets under lab conditions

Regarding the pupal weight, in general it decreased from the first three days (Supplementary Information, Table I), and then only slightly as the intrapuparial stage progressed (Fig. 5). There were differences between the sexes in both species: male *C. megacephala* pupae being heavier than female pupae for both diets (*p* < 0.001). On the other hand, female *L. sericata* pupae were heavier than male pupae for both diets (*p* < 0.001). On the last day of pupation for each diet, the male and female *C. megacephala* pupae had similar weights, but that was not the case for *L. sericata* (Supplementary Information, Table I). Considering the weight of each species in relation to the rearing larval substrate, *L. sericata* pupae were heavier when reared on minced pork meat than liver (*p* = 0.029). The same occurred for *C. megacephala* pupae, but the difference was not statistically significant (*p* = 0.127; Fig. 5).

### Larval competition experiment

In pure cultures, high densities reduced the mortality of *L. sericata* and *C. megacephala* (*p* < 0.001); however, the same did not occur in mixed cultures, perhaps due to the presence of the other species (Table 3). Specifically, the presence of *L. sericata* reduced the mortality of *C. megacephala* at the lowest density of 50 (*p* < 0.001) but increased it significantly at the highest initial larval density of 500 compared with pure cultures (*p* < 0.001). For *L. sericata*, mortality was not affected in the presence of interspecific competition (Table 3); it only increased slightly compared with the pure cultures when the density was high, that is to say at 200 and 500, but there were no significant differences at any of the densities (*p* > 0.001). The minimum mortality rate due to the presence of *Chrysomya* was never < 40%, probably due to the scarce medium larvae provided. The mortality of *C. megacephala* was high for both intra- and interspecific competition.

**Table 3:** Mortality rates (average ± standard error) and total minimum immature period from larva to adult (days ± standard error) at 23ºC for *Chrysomya megacephala* and *Lucilia Sericata* with intraspecific and interspecific treatments

**Density**	**Mortality (%)**		**Duration (days)**	
	**Intraspecific**	**Interspecific**	**Intraspecific**	**Interspecific**
	*Chrysomya megacephala*			
50	95.60 ± 0.75^a^	40.00 ± 4.00^a*^	15.40 ± 0.24^a^	14.00 ± 0.00^a*^
100	46.80 ± 5.41^b^	50.80 ± 3.26^a^	13.20 ± 0.20^b^	13.00 ± 0.00^a^
200	40.50 ± 2.29^b^	48.00 ± 8.43^a^	12.00 ± 0.00^c^	12.00 ± 0.00^a^
500	49.20 ± 4.78^b^	72.80 ± 3.20^b*^	11.00 ± 0.00^d^	11.00 ± 0.00^b^
	*Lucilia sericata*			
50	45.60 ± 4.66^a^	35.20 ± 3.20^a^	17.60 ± 0.24^a^	16.60 ± 0.24^a^
100	40.20 ± 2.84^a^	39.20 ± 5.24^a^	16.20 ± 0.37^ab^	16.00 ± 0.00^a^
200	25.70 ± 3.14^b^	32.00 ± 4.84^a^	15.20 ± 0.37^b^	15.00 ± 0.00^b^
500	29.88 ± 5.13^b^	32.80 ± 3.55^a^	16.00 ± 0.00^b^	15.00 ± 0.00^b*^

^abc^ Different letters between densities (within rows) or ^*^ between intra and interspecific treatment at same density (within columns) indicate significant differences *p* < 0.001

Regarding the life cycle span (Table 3), only data from larva to adult were used because in interspecific competition only adults were identified once emerged, and the first adults of each species were associated with the data for the adult stage. In general, as the density increased, the life cycle time decreased for *C. megacephala* (*p* < 0.001); the effect was less pronounced for *L. sericata*. There was a significant interspecific competition only at the lowest density for *C. megacephala* (*p* < 0.001) and the highest density for *L. sericata* (*p* < 0.001), where there was a reduction in the life cycle of both species.

## Discussion

*Chrysomya megacephala* has been confirmed as an early coloniser of vertebrate carrion and an important indicator species in forensic investigations [23, 27, 32, 46]. In general, this species is defined as synanthropic and inhabits areas with tropical and temperate climates. However, with current trends in global climate change, it seems likely that *C. megacephala* is colonising Europe and, therefore, could occur all over the world wherever the climate is sufficiently hot and moist [20, 47], as observed for *C. albiceps* on this continent [48]. Records of *C. megacephala* in the Iberian Peninsula [3–6] have increased in recent years. Our study is the first to report biological information on *C. megacephala* from a European population. We obtained the life cycle, with details of the period of each larval instar and stage, growth curves, as well as isomorphen diagrams; the LDT; and the ADDs – needed as tools in forensic cases – under constant and controlled laboratory conditions with a local population from Alicante (south-east Spain). Until now, the entomological evidence in south-west Europe has been analysed based on data from other geographic areas, with some exceptions [49–52]. However, the extrapolation of the data should be considered with caution, due to the variations between populations of the same species with wide distributions, such as *C. megacephala* [53] and *L. sericata* [10, 41]. We observed the effects on the behaviour and development due to population origin in *C. megacephala*, thus indicating the need for caution when using data from other regions. Our data, together with research from Egypt [54], should be used with caution in inland Europe. At present, it could be crucial for future forensic investigations involving corpses colonised by this species in southern Europe.

*Lucilia sericata* has different behaviours in northern and southern Europe: it acts as a myiasis agent in flystrikes and has a necrophagous habit in carrion, respectively. Moreover, researchers have observed differences in biological parameters in this species, such as the size of adults and the mortality rate depending on the origin of the population, probably with genetic population implications due to differences in hybrid specimens [10]. Some biological data of *L. sericata* for use in forensic entomology are available for Europe [8], but our study is the first to use a Spanish population. Nevertheless, our results are preliminary and limited, and studies including other constant temperatures are needed to confirm observation, and acquire thermal summation development models, as suggested by Richards and Villet [55].

*Chrysomya megacephala* and *L. sericata* are typical thermophilic species and are associated with warm climates [3, 34]. The members of *Chrysomya* are originally from tropical areas and can withstand high temperatures [23]. On the other hand, the greatest diversity and widest distribution of *Lucilia* species are concentrated in the north hemisphere, so it is considered a Holarctic genus. Regarding the interaction between the two species in the field, both species peaked in the months with an average temperature of 23 °C, and they coexisted in the same locations during the year. The maximum adult activity was in spring for *L. sericata* and at the beginning of autumn for *C. megacephala*, when humidity was high, due to the beginning of the usual Mediterranean rains after summer. Both species avoided the heat of summer, which has the highest temperatures during the year. *Lucilia sericata* basically peaks in summer at northern latitudes [56, 57] and mainly in spring in southern Europe, because the highest maximum temperatures usually occur in summer (as is the case for Spain). In Thailand, *C. megacephala* is more abundant when temperatures are higher, that is, in summer, and its numbers gradually decline during the rainy season and winter [58]. In India, *C. megacephala* increases at the beginning of the rainy season, and then declines in the dry, hot season [57]. In Brazil, the greatest abundance occurs between the end of spring and the end of summer, reaching a peak in the hottest month of the year [59]. Finally, in Egypt this species is captured mainly in spring and summer [32, 54]. This information indicates that the month of highest abundance depends on the geographic location and population changes based on the climatic conditions. In tropical countries, temperatures are practically constant during the year, but in Spain, there are unique meteorological conditions for each season, and the change between each season is relatively drastic. In Europe, the oriental latrine blowfly is a new species recorded in the Diptera sarcosaprophagous community: it is mainly captured in autumn, when other necrophagous blowflies such as *L. sericata*, *C. vicina*, *C. albiceps* (the most important facultative predator in the Mediterranean region), and the Muscidae species *Synthesiomyia nudiseta* (van der Wulp, 1883) are starting, reaching their peak, or finishing their activity [3, 11, 60]. The results support its status as one of the most globally relevant insects in medical and veterinary entomology [23], but also from the point of view of the ecology and biodiversity of the sarcosaprophagous Diptera community previously established in the European continent.

Our results confirmed the thermophilic character of *C. megacephala* and *L. sericata*, with similar physiology at high temperatures under laboratory conditions (3 and 28 °C), and slower development at 18 °C, with some differences. The size ranges for *L. sericata* and *C. megacephala* larvae we obtained are similar to previous studies, with a maximum of 14–17 mm [8, 61, 62] and 16–18 mm [21, 25, 27, 63, 64], respectively. In general, when temperatures increased, the larval size (length or weight due to both variables were correlated positively) increased and the duration of the stages reduced. *Chrysomya megacephala* larvae grew quickly, and without a stationary phase after peak feeding, while *L. sericata* larvae had a stationary phase, except at 18 °C, where the larval period was longer for both species. Moreover, the weight before pupation dropped markedly at 18 °C in both species, but also at 23 °C in *L. sericata*. The behaviour of *C. megacephala* at high temperatures, sometimes with mature larvae appearing on day 3, could indicate a better adaptation of this species to high temperatures compared with *L. sericata*. At high temperatures, *C. megacephala* could feed and then begin to migrate before other Diptera that colonise a corpse, such as *Lucilia* species; hence, there should be special attention regarding the moment entomological evidence is collected.

When we compared the life cycle and stages of *L. sericata* and *C. megacephala* with the published results from other geographic areas, we identified a similar pattern for both species. In general, there are differences in the development time at low temperatures, and it is long in the Spanish population [65, 66]. Regarding high and fluctuating temperatures, the differences are subtle [63, 67] and in *L. sericata* decreased as the temperature for comparison increased. It is worth mentioning the constant temperature of 18 °C, at which we observed significantly slower development compared with other studies. Sampling complete cohorts is essential to avoid sampling bias, to understand the variability in development rate [9], and to avoid the effect of depleting – which could have happened in this study at 18 °C, because we removed up to 80% of the larvae for our analysis – so the data in the present study at the low temperatures should be considered with caution [68]. In general, the results confirmed the hypothesis about the inherent biogeographic variation between blowfly population, mainly in *L. sericata*. The Spanish population appears to not be well adapted to low temperatures, so development in these conditions was slower than other northern populations that could be better adapted to cold periods [8, 66]. The development time was 26.7 days at 18 °C and 9 days at 28 °C for *C. megacephala*, with a longer duration of development of this species in the present study compared with other studies at low temperatures. For example, Goodbrod and Goff [69] obtained 9.8 days for the complete life cycle at 23.5 °C, and Milward-De-Azevedo et al. [65] confirmed that the development extends to 22.7 days at 18 °C. On the other hand, the results at higher temperatures are very similar to those found in other studies, such as that of Wijesundara [70] at 28 °C, who observed complete development in 8.2 days, and Ivorra et al. [27], who reported a total development time of 7.52 and 8.51 days at 32 and 27 °C, respectively. Other studies have provided additional data about the life cycle span of these species [23, 71, 72]. Our *C. megacephala* data confirmed the same abovementioned hypothesis about *L. sericata*: there is inherent biogeographic variation between *C. megacephala* populations. Finally, it is notable that neonatal *L. sericata* larvae were longest at the lowest temperature of 18 °C (3.42 mm), contrary to the trend observed for the length–temperature relation. This difference could be due to the larval development from the egg taking longer at low temperatures (in the present study, it lasted 2 days at 18 °C). Other authors have obtained similar results [71], but more studies are needed to determine the effect of low temperatures on the first hours of the egg and larval development, so the sampling frequency should be shorter (every 6 h) than in our study (daily) [55]. Gravid female flies could retain eggs for a long time in the absence of a suitable oviposition medium or unfavourable ambient conditions [73]. If temperatures are appropriate for maggot development (above the minimum developmental thresholds), then changes in environmental temperature do not seem to retard or alter maggot feeding [74, 75]. Thus, the first instar larval development in the egg could proceed, and the larvae emerging in these conditions could be larger. Nevertheless, in our study the lengths obtained under laboratory conditions (25 ± 2.49 °C) were congruent with the lengths obtained at constant temperatures, and similar to what could be expected based on growth curves at a constant temperature of 25 °C for both species. As it had been observed with *L. sericata*, the origin of the population could affect the life cycle span and explain these differences [53]. Therefore, more studies comparing different populations of *C. megacephala*, and with the same methodology and abiotic conditions, should be conducted.

Our evaluation of life cycle and the development at constant temperatures allowed us to obtain ADDs for both species. We calculated *t*_*0*_ for the first time for Spanish *C. megacephala* and *L. sericata* populations, obtaining a value of 12.78 and 9.78 °C, respectively. The value obtained for *C. megacephala* is higher than what has been obtained for populations from South Africa (*t*_*0*_ = 10.4ºC) [55], India (*t*_*0*_ = 10 °C) [76], the southern United States of America (*t*_*0*_ = 10.95 °C) [77], and China (*t*_*0*_ = 11.41 °C) [64], but similar to what has been obtained for a Sri Lankan population (*t*_*0*_ = 13 C) [73]. However, in the case of *L. sericata*, our data are consistent with those recorded by Marchenko [66] and Higley and Haskell [78], who estimated a *t*_*0*_ of 9 and 10 °C, respectively. In general, we found that development under fluctuating temperatures in the laboratory agrees with the development under the mean daily temperature and could be readily used to determine the developmental stage of any species located when development occurs at constant temperatures [78]. However, some necrophagous species develop at different rates under fluctuating temperatures [51, 79]. In the case of the tropical species *C. megacephala*, fluctuating temperatures and annual seasons, typical for temperate regions, have an unknown effect.

Several factors are known to affect the predictable relationship between the larval development rate, size, and temperature, and, consequently, the PMI estimation. Some researchers have also suggested that the tissues on which the larvae feed might also influence the growth rate [38, 71, 80]. Consistent with this conclusion, when we compared larvae based on the feeding substrate, in general the larvae that fed on minced pork meat were larger than the larvae the fed on liver. Moreover, the life cycle was shorter when fed on minced pork meat, but the differences were not always significant. The nutritional intake of larvae varies depending on the part of the corpse on which they are feeding. The maximum length was reached earlier on minced pork meat than on liver in both species. During the period that the larvae fed, the sizes registered on the liver were smaller than on minced meat. However, after reaching its maximum length, the size of the larvae fed on minced pork meat decreased, while the size of the larvae fed on liver stabilised or decreased, which resulted in larvae that were similar in size before pupation regardless of what they fed on. In fact, we observed that pupation occurred sooner on minced pork meat than liver in both species. These results demonstrated that growth on liver was slower than on minced pork meat, but larvae in the prepupal phase on minced meat lost more biomass than larvae on liver, or this phase was shorter.

In *L. sericata*, it has been observed that larvae reared on homogeneous tissues (such as liver or heart) are smaller than those reared on mixed tissues [81]. The tissue structure influences its breakdown, and larvae may take longer to feed [82]. White meat contains less connective tissue than red meat [83], so it is easier for larvae to consume minced meat. This probably explains why flies fed on minced pork meat were significantly longer compared with those fed on liver. Warren et al. [84] indicated that *L. sericata* larvae fed on pork substrates were smaller than larvae fed on beef substrates but caught up in size with those feeding on beef substrates with an extra day of feeding. Our observation of a minor loss in the prepupal phase and a longer feeding period in larvae reared on liver agrees with these authors. However, our findings differ from another study that used English populations of *L. sericata* [38], but this could be the result of geographically separate populations (English *L. sericata* is known for feeding mainly by myiasis on sheep) [10, 41, 42].

In the case of *C. megacephala*, development studies performed throughout the world have shown that the diet on which this species is reared at similar temperatures may cause it to require different amounts of time to complete its cycle of growth [71]. This difference is caused by the different food sources provided to the colonies and/or the ecological adaptation of the species to the different biogeographical and bioclimatic zones [85]. The eusynanthropic lineage of *C. megacephala*, which originated in New Guinea, is restricted to the densely populated urban and suburban areas of the world due to it being attracted to a wide variety of human foods, human and livestock faeces, and carrion [21]. This species is of forensic importance, and we have shown that the tissue on which it feeds affects its growth rate; hence the substrate used for laboratory rearing and subsequent investigation should be considered before drawing estimating the PMI.

Finally, the capacity to adapt to abiotic conditions in the intrapuparial stage was evident in both species. The weight of the pupa in both species was influenced by the larval diet and sex of the emerged adult, as the pupae were heavier on minced meat than liver; the female *L. sericata* pupae and male *C. megacephala* pupae were the heaviest. The intrapuparial periods were constant in all cases, but the male *C. megacephala* adults emerged 1 day before the female adults when fed on liver, indicating that liver is a worse larval diet than minced pork meat for rearing this species. This observation agrees with Picard et al. [86], so the sex of the emerged *C. megacephala* adult should be considered when estimating the mPMI from pupae collected in a forensic case. In summary, the type of tissue on which larvae feed affects the length of the larvae, and it could alter the PMI estimation; the position at which larvae were feeding on a body is a crucial observation at the scene of death. Usually, in the presence of wounds, larvae begin to feed on these tissues, where access to fat, muscle, and blood is easier [38, 81].

The implications of the presence of *C. megacephala* on the dynamics of the sarcosaprophagous community and its effect should be studied [87], so we conducted a larval density competition study with *C. megacephala* and *L. sericata* larvae in laboratory conditions and at high level of competition [60, 88]. In our experiment at a constant temperature (23 °C and *ad libitum* feeding), the period from egg to adult emergence was 13.2 ± 0.9 days for *C. megacephala* and 16.8 ± 0.8 days for *L. sericata*. These results are similar in the case of intraspecific competition for *C. megacephala* at an initial larval density of 100, but when the density decreased this period increased significantly, and when density increased the life cycle period decreased. Similar density-dependent behaviour has been observed in other carrion flies [60]. The interaction between species and population aggregation on carrion is an important factor to consider when estimating the mPMI in real cases [11, 89]. Under interspecific competition at the lowest density, *C. megacephala*’s lifespan and mortality decreased significatively with respect to intraspecific cultures because of the presence of *L. sericata*. Moreover, at the highest density, the mortality for interspecific cultures was higher compared with intraspecific cultures. In conclusion, at the lowest density the presence of *L. sericata* increased the survival of *C. megacephala*; however, at the highest density, the mortality of *C. megacephala* increased when *L. sericata* was present. This phenomenon could be related to our observations about the role of *C. megacephala* as a coloniser of corpses [90]. In the case of *L. sericata*, the lifespan was similar to that observed in previous studies [10], and at high densities it decreased in the presence of *C. megacephala*. In other competition experiments where other species fed together with *L. sericata*, such as *C. vicina*, the larval periods decreased [60, 90]. The mortality rate was not significantly affected, unlike what has been observed when this species coexisted with the predator *C. albiceps* [60]. Showing that adults are active at the same location and at the same time does not prove that both species oviposit on the same carcasses/corpses at the same time. However, the larvae have been reported to co-occur during succession in China [91]. In Spain, *C. megacephala* larvae have been collected with *L. sericata*, and they have been observed feeding on a pig cadaver (*Sus scrofa*, approximately 25 kg); in this case, *C. megacephala* larvae were found on cadaver around day 10 of exposure, and in different locations than *L. sericata* [36].

## Conclusions

This study provides the first data regarding the biology and ecology of *C. megacephala* and *L. sericata* from south-west Europe. Both species coexist, but their behavioural strategies seem to be different in the field and in the laboratory. Based on the results concerning the fast development of *C. megacephala*, and due to its plasticity and capacity to develop at a wide range of temperatures, as well as its feeding without problems on different substrates, this species has great potential to introduce itself into communities of sarcosaprophagous Diptera, which explains why it has spread around the world so successfully. It would be useful to study this species from biological and ecological points of view in Europe, so attention needs to be given regarding how representative it is in the Diptera carrion community and what its effects are on other blowflies. All ecological and biological data could be very useful in the resolution of forensic cases from Europe, where immature stages of *C. megacephala* and *L. sericata* coexist.

## Electronic supplementary material

Below is the link to the electronic supplementary material.

Supplementary Material 1

Supplementary Material 2

Supplementary Material 3

Supplementary Material 4
