# Supplementary material for: Dietary and competition effects on life history attributes of Chrysomya megacephala and Lucilia sericata (Diptera: Calliphoridae) in south-west Europe
Source: Int J Legal Med. 2025 Jan 23;139(3):1423–36. doi: 10.1007/s00414-025-03425-1 (PMC12003503; doi:10.1007/s00414-025-03425-1)
Supplement: Supplementary file 4 — Supplementary Material 4 [file 414_2025_3425_MOESM4_ESM.docx]

**Figure I.** Isomorphen diagram for *Chrysomya megacephala* and *Lucilia sericata.* Lines represent a morphological change: egg hatching (E1), first moult (E2), second moult (E3), development of puparium (P) and adult emergence (A). Area between lines means (± standard error) duration of egg, first instar, second instar, third instar and pupa.

MS Office was used to create the figure
